# Epigenetics and Congenital Heart Diseases

**DOI:** 10.3390/jcdd9060185

**Published:** 2022-06-09

**Authors:** Léa Linglart, Damien Bonnet

**Affiliations:** 1 M3C-Necker, Hôpital Universitaire Necker-Enfants Malades, Assistance Publique-Hôpitaux de Paris (AP-HP), 75015 Paris, France; lea.linglart@aphp.fr; 2 School of Medicine, Université de Paris Cité, 75006 Paris, France

**Keywords:** congenital heart disease, epigenetics, genetics

## Abstract

Congenital heart disease (CHD) is a frequent occurrence, with a prevalence rate of almost 1% in the general population. However, the pathophysiology of the anomalous heart development is still unclear in most patients screened. A definitive genetic origin, be it single-point mutation or larger chromosomal disruptions, only explains about 35% of identified cases. The precisely choreographed embryology of the heart relies on timed activation of developmental molecular cascades, spatially and temporally regulated through epigenetic regulation: chromatin conformation, DNA priming through methylation patterns, and spatial accessibility to transcription factors. This multi-level regulatory network is eminently susceptible to outside disruption, resulting in faulty cardiac development. Similarly, the heart is unique in its dynamic development: growth is intrinsically related to mechanical stimulation, and disruption of the intrauterine environment will have a direct impact on fetal embryology. These two converging axes offer new areas of research to characterize the cardiac epigenetic regulation and identify points of fragility in order to counteract its teratogenic consequences.

## 1. Introduction

Congenital heart disease (CHD)—structural defects in heart morphology—is one of the most-represented types of congenital anomalies, with a prevalence of 1% in the general population [1]. Despite the wide array of potential mistakes, the spectrum of observed phenotypes in patients intriguingly converge in a finite set of entities [2]. After multiple systems of patient classification, the most logical and medically appropriate emerged from heart embryology: the various phenotypes we observe in CHD can be easily related to embryological errors, and precise knowledge of the sequential events and spatiotemporal interactions between heart substructures is intrinsic to understanding pathophysiology [3].

With the great genetic revolution came the hope that unlocking the molecular bases for CHD would provide fluid gene/disease correlations; this sadly was far from the case in the domain of congenital cardiac diseases. Today, with the advances in genome annotations and mass sequencing of patient DNA, only 35% of all occurrences can be clearly linked to a genetic origin, be it single-gene mutations (3–5%), aneuploidies (8–10%), or copy number variants (3–25%) [4]. Even in the case of identified genetic substratum, the mutation/disease model remains inapplicable, as is evidenced by (1) the convergence of various mutations to identical phenotypes; (2) conversely, the multiplicity of phenotypes associated with a single identified mutation; and (3) the incomplete penetrance of these mutations [5]. The key to understanding the anatomical spectrum of CHD therefore lies in epigenetics, a concept here encompassing all modifiers external to the genetic code in itself, affecting the temporality and level of expression of these genes as well as modifying environmental factors.

Epigenetic regulation is multidimensional in nature, and plays a crucial role in heart organogenesis, as evidenced by the high prevalence of CHD in syndromes involving epigenetic regulator mutations: histone-modifying genes alone are estimated to contribute to 10% of CHD patients [6]. Ergo, disruption of these regulatory mechanisms offers a convincing mechanism for explaining CHD prevalence and polymorph presentation within an identified genetic background, as suggested by the numerous examples of divergent monozygotic twins [7,8]. Intrinsically, heart embryology is shaped by interactions with its environment, and particularly with hemodynamic signalization, making it both specifically vulnerable to aberrant development in response to external hemodynamic stimulus; but also conferring it a high degree of adaptability, hereby explaining the finite spectrum of phenotypes encountered as viable up to birth. The highly complex and multiple regulatory levels in genetic regulation make it eminently difficult to unravel the precise mechanism in which epigenetic regulators—maternal environment, hemodynamic variations, micronutrient availability, toxicant exposure—mediate embryological errors. However, as data accumulate, these factors could bridge the gap between identified and unexplained cardiac pathogenesis and allow for potential protective intervention.

## 2. Morphogenesis, Embryology and Disease Spectrum

Morphogenesis of the heart is established very early on in organogenesis, between 15 and 45 days after conception. The emergence of a functional circulatory system is crucial to the survival of the embryo, as tissular diffusion alone becomes unable to cope with the rapidly increasing size of the embryo [9]. At day 30, most major cardiac structures have been established in their spatial conformation; further remodeling will occur later on at a smaller scale in tissue anatomy, with the development of the supporting vasculature and the maturation of heart trabeculations being the closing points on organ development [10].

Heart progenitors can be identified from day 15 onwards, localizing in a crescent-shaped region known as the first heart field (FHF) [11]. Medially to this heart crescent, a second area, the Second Heart Field (SHF), will also contribute to heart morphogenesis through a mechanism of addition, building throughout cardiogenesis upon the scaffold established by the FHF skeleton [12]. The FHF undergoes fusion at the midline, establishing a primitive linear cardiac tube. Spatial localization within the tube (and ergo the loop) is intrinsically linked with cellular fate and function: cellular identity and organ asymmetry are established in a two-dimensional axis in the first phase of development (left/right lateralization) within the crescent-shaped heart fields and fine-tuned in three-dimensional spatial regulation at the tube-formation phase [11]. Throughout the subsequent steps of cardiogenesis, proper development will be wholly reliant on effective crosstalk between the various cellular progenitors, including FHF, SHF and neural crest cells [13]. Errors in cellular migration, cellular interaction, regulated apoptosis and proliferation will affect the alignment of structures, tissular growth and the resulting flow pattern throughout the developing organ, resulting in the wide pathological spectrum of CHD [14,15,16].

A functional, beating heart is indispensable for embryogenesis to pursue, and anomalies affecting cardiac structure result in a dichotomous outcome: viability or spontaneous abortion. A high number of spontaneous miscarriages are thought to result from unviable cardiac phenotypes [17]. The genetic cascades activated throughout heart development are exclusively fundamental cellular pathways: cell proliferation and apoptosis, cell migration and embryo lateralization. High-impact point-mutation on such primitive signalization pathways would never result in viable pregnancies, explaining the low percentage of identified point-mutation in CHD cohorts, and seemingly implicates cardiac specific transcription factors in many cases: GATA4-6, NKX2.5, TBX1, NOTCH, JAG to cite a few that are frequently identified in genetic screening of patient cohorts [18]. It can be postulated that hits on these molecular “switches” could potentially be bypassed with parallel redundant cascades, allowing for potential “rescue”—hence explaining the variability of phenotypes stemming from identical point-mutations and once again introducing the concept of “modifiers”—i.e., epigenetics. Genome-wide analysis is now conceptualized as veritable networks of genes, each node influencing and regulating multiple adjacent factors [19]: the regulation of expression, more than the gene product itself, becomes a potential answer in understanding CHD.

## 3. Epigenetic Alterations in Heart Disease

**Figure 1:**
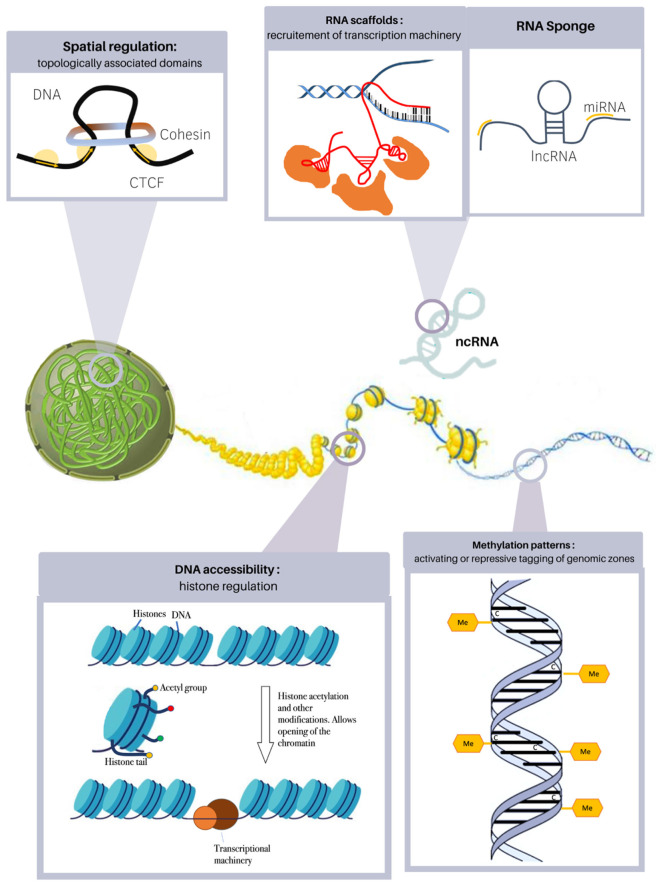
Epigenetic regulation mechanisms involved in heart morphogenesis.

In recent years, multiple levels of gene expression regulation have been discovered and explored; mutations on these regulatory elements understandably result in multisystemic involvement. Congenital heart defects are not spared from this, and both in vivo and patient observations have shed light on the major role epigenetics play in CHD (Figure 1).

Within the nucleus, excluding the tightly packed chromosomal state of mitosis, DNA resides as an unwound double-strand: the DNA molecule is arranged in specific conformations, within spatially defined domains. Within these domains, regulatory elements and multiprotein complexes act as scaffolds for bringing in contact distant genomic regions for combined temporospatial expression. These zones are known as topologically associated domains and allow for co-regulation of genetic targets [20]. Establishment of TAD is under the control of architectural proteins such as CTCF, which delimitates the borders of topological domains. Cohesin complexes also play a role in the dynamic function of TADs: this circular protein structure is loaded unto the DNA strand and will progressively extrude the chromatin into expanding loops until they run into insulator proteins (CTCF), bump into each other or dissociate from the molecule, effectively bridging together distant genomic regions [21]. Disruption of these regulatory mechanisms, as evidenced by many examples in pathophysiology, will directly result in CHD phenotypes. Cornelia de Lange syndrome, resulting from mutations within the cohesin complex (NIPBL, HDAC), frequently manifests with CHD, TOF in 50% of cases, VSD, ASD, PDA and valvular abnormalities [22].

Accessibility of transcriptional machinery to its target sites will depend on the local “openness” of the genetic information. The DNA filament is packed around protein units, an octamer of histones organizing the chromatin in functional units known as nucleosomes. The nucleosomes will directly regulate how tightly DNA is packed through electrostatic interaction with the adjacent histones and DNA itself. To effectively open and close chromatin, the histone tails are modified through acetylation, phosphorylation and deacetylation; these tags may play a permissive or repressive role in gene accessibility [23]. Mutations in SMAD2, a regulator of H3 methylation, were shown to be excessively represented in Whole-Exome analyses comparing CHD versus unaffected subjects [6]. T-box proteins, mutated in Holt-Oram syndrome (TBX5) and DiGeorge syndrome (TBX1), display highly conserved residues, allowing direct interaction with both histone demethylase (H3K27) and methyltransferase (H3K4): both syndromes include cardiac manifestations [24], underlining the highly regulatory nature of these pathogenic mechanisms; treatment with demethylase inhibitors of Tbx1-KO mice rescues the cardiac phenotype [25]. Histone acetyltransferase variants were shown to be associated with CHD (ventricular septal defects, atrial septal defects, patent ductus arteriosus and tetralogy of Fallot) in a Chinese Han cohort [26], perhaps hinting at a mechanism for polygenic susceptibility models. Histone modifications also have the advantage of being highly dynamic in nature and allowing time-specific regulation of gene expression: PRDM6, a methyltransferase involved in maintaining cells in an undifferentiated stage with proliferative potential is highly expressed in ductal tissue, and will drastically fall in the postnatal phase, allowing for differentiation and ductus arteriosus closure. Disruption of its activity results premature differentiation and persistent ductus arteriosus [27].

Once the chromatin has been established as open and accessible, direct DNA methylation can once again orient transcription profiles by restricting or priming anchorage of transcription machinery. Addition or removal of methyl groups to nucleotide regions by methylases and demethylases is an extremely dynamic and fine-tuned way of adjusting the accessibility of genic domains by impeding attachment of transcription factors or gene-expression protein complexes. Multiple studies have proven differential methylation, both at the genome-wide level and specific coding regions, in CHD-affected patients [28,29,30]. Even more specifically, different methylation patterns are observed within discordant monozygotic twins for tetralogy of Fallot and the double outlet right ventricle—although the global genome-wide methylation burden does not differ, specific promoters are highly divergent in CpG marking, and can be linked back to cardiac embryology (TBX20, NFATC1 involved in valve formation, GATA4, NKX2-5, NOTCH4) [31,32]. Methylation plays a crucial role in embryology: cells undergo a widespread wave of demethylation to reestablish pluripotency during fertilization. As differentiation progresses, embryologic cascades seem to be progressively switched off through inhibiting methylation (hereby protecting the organisms from unregulated proliferation and cancerous predisposition) [33]. Conversely, targeted regions in specific cell precursors are actively kept in a demethylated state, in veritable cell-specific patterns, making them rapidly available for future activation. Knockout of demethylation enzyme TET1 in vivo recapitulates this mechanism, as affected cells display inhibitory hypermethylation at the NKX2-5 promoter, to cite one of many, effectively blocking cardiomyocyte differentiation [34]. This allows the future identity of yet-undifferentiated progenitors to be established at the very initial steps of heart development.

Finally, recruitment of multiprotein complexes interacting with DNA in all of these stages upstream of actual gene expression—methylation, histone modification, DNA conformation modification—can be in itself regulated by regulation molecules. Non-coding RNAs are emerging as prime candidates for this trans-acting modification: acting as scaffolds for machinery assembling and targeting, or sponges for dosage regulation of the transcribed RNAs, they add another intermediary step before protein expression which can be influenced by outside modifiers. Variations in levels of non-coding RNA have been explored in multiple studies and provide further support to their implication as regulators: targeted knockdown of cardiac-specific lncRNAs such as Handlr and Atcayos proved in vivo interaction with crucial cardiac nodes such as HAND2 and BMP4 [35]. In bicuspid aortic valve patients, miR-29 seems to be specifically downregulated [36]. However, as underlined by George et al., ncRNAs seem to have modest and easily bypassed impacts on cell fate, and so far have not been linked to CHD in large-scale screens [35].

Through this quick recapitulation of the principal epigenetic regulations of gene expression, it appears evident that these multiple steps are only so many weak points potentially affected by outside influences in the course of organogenesis.

Another interesting emerging hypothesis is the new outlook epigenetics gives us on large-scale chromosomic aberrations. Stepping away from the established concept of establishing parallels between manifestations of a syndrome and the associated deleted/overrepresented genes in linear pathogenic linkage—for example, DiGeorge syndrome and TBX1 deletion included within the 22q11.2 deletion explaining the cardiac involvement—the syndrome could effectively be classified as regulopathy, as for each example, crucial DNA regulation genes are involved and have consequences that have rippling repercussions on more than the affected regions. Down syndrome epigenetic studies have proven that the surnumerous chromosome results in differential expression with upregulation of 27% of genes on chromosome 21, 4.4% of genes on other chromosomes [37]; a concordant study found misregulation of 247 genes not located on chromosome 21 [38]. This is thought to be due to the additional copy of DNMT3L, a methyltransferase present on chromosome 21. In the case of Turner syndrome, haploinsufficiency of KDM6A—a histone demethylase mutated in Kabuki syndrome and known to play a crucial role in cardiac embryogenesis—is thought to be one the mediating elements in the development of heart defects [39]. Epigenetics may turn out to be the basis of the majority of syndromic cardiac pathologies (Table 1).

## 4. Environmental Slights

**Figure 2:**
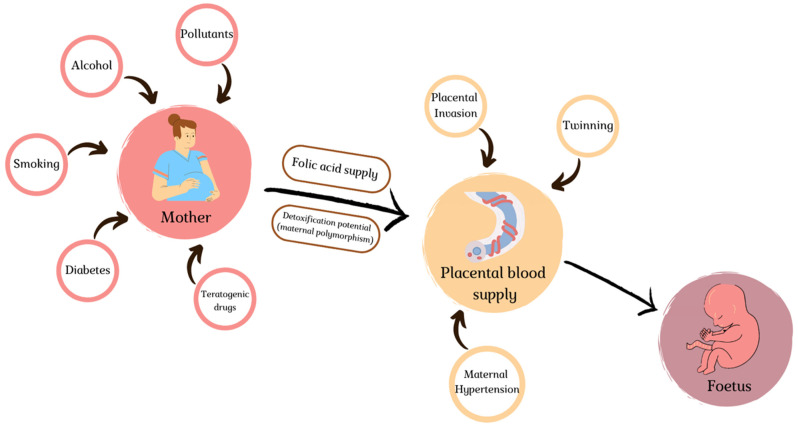
External influences with teratogenic potential in fetal and cardiac development. The mother/placental/fetal axis is subject to numerous external environmental slights at its different levels.

Cardiac embryogenesis seems eminently susceptible to outside aggressions in the earliest stages of fetal development (Figure 2), as organogenesis relies on interaction with various cellular populations to induce remodeling in a precise temporospatial sequence and responds to flow patterns to modulate its development. This dependency on external signalization implies possible disruption of the morphogenetic signals.

Twin pregnancy has been established as an independent risk-factor for CHD, estimated at a 60% risk increase in a large-scale Danish cohort [52]: chorionicity seems to be the principal risk situation, with monochorionicity resulting in a nine-fold increase in CHD [53]. This hints at a pathophysiological mechanism linked to abnormal placentation; the high occurrence of discordance in CHD presentation within the twin pairs suggest unequal partition of blood flow, ranging from simple arteriovenous anastomoses with unidirectional blood flow to the pathological condition known as twin–twin transfusion syndrome. In the former, despite the absence of TTS, the low-level circulatory imbalance will still be hemodynamically detectable with increased aortic and pulmonary velocities in recipient fetuses, and result in a seven-fold increase in the risk of structural defects [54]. In this latter case, blood flow is preferentially addressed from the donor to the recipient fetus, with grave hemodynamic alterations in both subjects, and a 13-fold increase in CHD risk [53]. The donor fetus often presents with cardiac anomalies related to the insufficient hemodynamic load, valvular stenosis and secondarily hypoplastic ventricles [8]; coarctation of the aorta has also been observed in donors, in which the hypoplasia results from insufficient prenatal blood flow through the aortic arch [55]. Meanwhile, the recipient responds to the increased preload by activating neurohormonal renin–angiotensin systems, flooding both circulation with neurohormonal mediators and affecting blood perfusion even more; CHD will principally manifest as atrioventricular regurgitation and right-sided outflow tract obstructions [56]. Intrauterine intervention will improve cardiac function in severely affected fetuses, but post-interventional observational studies showed an unchanged occurrence of CHD [57]; this observation seems concordant with the hemodynamic slight occurring during crucial steps of embryology, resulting in anatomical defects, and suppressing the anastomoses between twins will only rescue the pathological load associated with post-morphogenetic heart adaptation. In vivo studies on model organisms have already shown the direct effect of fluid alteration on organ development in crucial pathways [58,59,60]: atrioventricular septation is dependent on local shear-stress, as the mechanical stimuli will be translated into activating signal for the valvulation processes [61]. Similarly, growth of various cavities and appropriate trabeculation of ventricles is directly dependent on the pressure exerted on walls [62].

In another spectrum of morphologic anomalies, twins seem to be at a higher risk of CHD unrelated to hemodynamic load, and specifically for laterality disorders. In the case of monochorionic pregnancies, which seem to bear the highest risk of CHD development, blastomere division occurs at 3–9 days in the case of monochorionic diamniotic twins, and even later for monochorionic monoamniotic twins: around 9–12 days (vs. less than 3 h for dichorionic pregnancies) [63]. When considering the epigenetic signaling referred to above at the earliest stage of organogenesis, i.e., cellular priming through methylation profiles and laterality establishment within the heart tube, division of the cell mass will inevitably result in unequal partition of cellular components for the future organ [64]. Twin pregnancies present a five-fold increase in risk of heterotaxia, an otherwise rare occurrence in the spectrum of CHD (1/24,000 live births) [56]. Authors have gone so far as to impute singleton heterotaxia to the loss of an undiagnosed monochorionic twin.

Even in the context of singleton pregnancies, hemodynamic supply (and its alteration) seems to be a major physiopathological mechanism for aberrant heart development. Optimal oxygenation of the fetal unit requires regulated placental invasion and the development of a low-resistance, high-surface vasculature to allow nutrient diffusion. Hypoxic insult to cardiogenic precursors in the SHF was shown to correlate with the occurrence of CHD in a linear severity pattern [65]. Observational studies on cohorts of CHD-affected subjects showed significant variations in placental development, both in terms of volume and micro-architecture [66]. Abnormal invasion results in preeclampsia, with a seven-fold risk increase observed in pregnancies complicated by heart defects [66]. Cord anomalies are particularly frequent in CHD, with a study citing a 13% incidence of fetal heart defects in single umbilical artery subjects [67], and similar overrepresentation of anomalous insertions in CHD cohorts (eccentric, marginal and velamentous insertions) [68]. Embryologically speaking, this association is referred to as the placenta–heart axis: these organs develop at identical timepoints and are the earliest necessary to pursue embryonic growth [69]; disruption on either side of this axis will result in aberrant morphological development, as evidenced by the microvasculature anomalies observed in CHD pregnancies, encompassing both maternal malformations (maternal vascular malperfusion, resulting from aberrant implantation) and fetal venous malperfusion (modifications in vasculature resulting from hypoxic/polyglobulic return to the placental unit in the context of cyanotic CHD) [68,69]. Overactive immune response or maternal toxicants impairing effective placentation may contribute significantly to CHD [70].

Certain maternal comorbidities directly link back to this hemodynamic instability affecting fetal development: hypertension predating pregnancy has been identified as a clear risk factor for congenital heart defects in large-scale cohort studies, with an estimated risk increase of 50–60% [71]. Interestingly, this risk is even higher in treated mothers (RR 2): it has been suggested that antihypertensive medication would have intrinsic fetal hemodynamic repercussions, impeding appropriate blood flow to the developing heart cavities [72].

Diabetes and obesity, which are also on an upward trend in women of childbearing age, similarly confer an added risk of CHD, but the pathophysiological insult here is more biochemical than hemodynamic in nature. Pregestational diabetes, implying exposure of the embryo to hyperglycemia at the very earliest period of development and, ergo, during heart organogenesis, results in a three-fold increase in CHD [73]. The spectrum of observed phenotypes supports early hits on the embryological development as translated by the highly teratogenic effect (transposition of the great arteries, persistent truncus arteriosus, heterotaxia, single ventricle) [74]. High-level glucose exposure of chick embryos recapitulates these teratogenic findings [75], and alarmingly, even moderate elevations in HbA1c confers elevated risk, a cutoff of 9% being sufficient to predict risk [76]. Hyperglycemia has been shown to impede cellular migration and deregulate apoptosis and proliferation signaling [77]; chronic exposure also seems to induce overexpression of enzymes catalyzing the production of reactive oxygen species, overall favoring high-level oxidative stress [76]. In the case of obesity, in which we observe a linear association between BMI and CHD prevalence [78], one must consider the epidemiologic association with undiagnosed diabetes and insulin resistance, which contributes significantly to the observed risk. Another crucial parameter lies in folate availability: as will be broached later, folate deficiency, which is the cornerstone of epigenetic pathogenesis, is particularly frequent in overweight mothers, and general-population supplementation may be insufficient to obtain appropriate levels at the embryological level [79].

Folate bioavailability is a crucial factor in embryo development. Sufficient levels are necessary for DNA synthesis; it also plays the role of methyl donor in previously mentioned epigenetic regulation mechanisms (DNA methylation, histone modification) [80]. Folate deficiency has a direct impact on genetic regulation, as evidenced by the global hypomethylation levels observed in offspring of folate-deficient mothers [81]. Similarly, hypomorphic polymorphisms in folate-cycle enzymes such as MTHFR (methylenetetrahydrofolate reductase) [82] or MTRR have been established as risk factors for cardiac defects, and directly linked to DNA methylation levels [83]. Conversely, maternal supplementation with polyvitamins in the periconceptional period effectively rescues this phenotype, with an estimated drop in fetal risk of 30% [84]. Even more strikingly, this effect has been observed at a nationwide scale, as developed countries initiated folate fortification of foodstuffs: in Quebec, implementation in the 1990s resulted in a 6% drop per year in CHD occurrence [85]. Inappropriate bioavailability for embryos could recapitulate the higher prevalence observed in low-income and malnourished populations, obese mothers and even twin pregnancies [86]. It is important to note that despite a globally lower methylation status of DNA, specific loci in deficient mothers display pathological hypermethylation [81], hinting at a global disruption of epigenetic regulators.

Another potential source of epigenetic dysregulation lies in fetal exposure to toxic compounds: multiple compounds disrupt gene expression and result in cardiac defect phenotypes.

Amongst the numerous identified teratogenic drugs, some are known to directly and specifically perturb heart organogenesis. Thalidomide, initially used as antiemetic medication in pregnant women, was quickly shown to directly impact cardiogenesis with a phenotype curiously recapitulating the defect found in Holt-Oram syndrome (caused by TBX5 mutation) involving CHD—particularly VSD—and limb malformation [87]. Analysis of the molecule quickly uncovered a mechanism of direct linkage of the drug with TBX5 in vivo, effectively impeding attachment of the transcription factor to target loci [87]. Another oft-cited drug is Lithium, a widely used neuroleptic, with exposed offspring being particularly susceptible to Ebstein’s anomaly with inadequate delamination of the tricuspid valve [88]. The physiopathological mechanism lies in the inhibition of the enzyme GSK-3 (glycogen synthase kinase 3), mimicking Wnt-signaling in doing so, signaling which must be repressed in the earliest stages of heart organogenesis to allow endothelial to mesenchymal transition, cell proliferation and migration [89]. Valproic acid, used as an antiepileptic medication, similarly has a direct repressive effect on GSK3 and in utero exposure causes major cardiac malformations including valve atresia and double outlet right ventricle [90]. Valproic acid is also known to act as a direct inhibitor of histone deacetylase 3, thereby widely deregulating gene control throughout the genome [91]. Most interestingly, in both lithium and valproic acid exposure, folic acid supplementation rescues the cardiac phenotype in animal models [89], and high-dose supplementation is now recommended in all epileptic women before pregnancy [92].

Environmental exposures can have deleterious impacts on fetal development: despite methodological difficulties in accurately determining the level of exposure of pregnant mothers and the associated risk increase in offspring, numerous gaseous pollutants, pesticide byproducts, heavy metals and numerous others have been linked to CHD risk. Trichloroethene, a halogenated hydrocarbon contaminating water sources, increases risk of cardiac malformation by reducing the expression of nitric oxide synthase (hereby exposing the embryo to higher levels of free radicals), disrupts epithelio-mesenchymatous transition of valve progenitors, and disrupts VEGF proliferative signaling [93]. Cohorts evaluating the impact of exposure to high concentrations of agricultural pesticides in 300,000 pregnant mothers found higher incidence of CHD, with a particular risk for atrial septal defects (ASD–OR 1.7) and patent ductus arteriosus (PDA–OR 1.5). Exposure to solvents in the periconceptional time-period similarly conferred an increased risk of ventricular septal defects (VSD), outflow tract obstruction, pulmonary stenosis [94]. Dioxin exposure in gestating mothers results in increased incidence of cardiac malformation, as underlined by the identification of a cluster of hypoplastic left-heart syndromes in Baltimore [95]: this compound was found to directly impact cardiomyocytes differentiation by reshaping genome methylation through downregulation of the methyltransferases Dmnt3a and 3b [96]. Finally, air-pollutant exposure, elaborated on in the 2016 review by Vecoli et al., can also expose fetuses to important inflammatory stress: SO2, a particularly oxidant chemical, was associated with occurrences of VSD; elevated CO concentrations, dissolved in plasma into high levels of carboxyhemoglobin, directly induces hypoxia at the fetal level; fine-particle exposure has been correlated with pulmonary valve stenosis, with an OR of 2.6 for the fourth quartile [97]. Although nowadays, there are too many exposures to properly investigate, it appears significant that all environmental pollutant intoxications seem to induce cardiac manifestations, underlining the intense vulnerability of heart development to outside perturbators.

More easily quantifiable is direct maternal sources of toxicant exposure, i.e., smoking and alcohol consumption. Both firsthand [98] and secondhand [99] smoke exposure increase CHD occurrence in a multifactorial process involving microcirculatory events and the hemodynamic alterations induced; inflammation cascade activations; carboxyhemoglobin; and resultant fetal hypoxia [100]. In vitro, tobacco smoke was shown to directly impact Gata4, Nkx2-5 and Mef2c activation (essential cardiogenic cascades) through hypoacetylation of the Gata4 promoter [98]. Regarding alcohol consumption, precise characterization of the associated risks is crucial, as a single high-level intoxication in the earliest stages of pregnancy—i.e., oftentimes before the pregnancy is known—will coincide with heart embryogenesis and have irrecuperable effects. A wide spectrum of cardiac defects is associated with prenatal alcohol exposure, including VSD, ASD, atrio-ventricular septal defects (AVSD), conotruncal defects, pulmonary valve stenosis [101]. Experimental models with high-dose embryo exposure to ethanol have recapitulated this wide array of presentation [102]: the embryological impact seems to be mediated by both alteration in retinoic acid signaling, and profound disturbance of histone regulation with hyper-H3K9 acetylation through activation of histone-acetyl-transferases [103]. Ethanol exposure also appears to target CNN derivatives specifically, explaining the increased prevalence of conotruncal pathologies [104]. Current efforts in protective supplementation, similar to folic acid in the case of valproic acid toxicity, have shown potential reversal of alcohol exposure with glutathione (the “master antioxidant”) administration, but hopes of preventing the cardiac phenotype would imply prenatal continuous administration [102].

The last element to consider with the changing landscape of periconceptional research is the increasing importance of Assisted Reproductive Technologies, which have been shown to confer a 30 to 40% increase in the risk of CHD without chromosomal abnormalities [105,106]. This association remains significant after correction for twinning and seems to be more consistently observed in IVF and ICSI techniques than in ovarian stimulation and intrauterine insemination, implying a pathogenic effect of direct in vitro gamete manipulation [107] (cryopreservation, use of varied culture artificial media, mechanical manipulation). In a cohort of Beckwith–Wiedemann patients, methylation patterns significantly differed between patients born via ART versus patients conceived naturally [108]. As established earlier, cell fate is determined very early on epigenetically, even before activation of specific transcriptomic profiles, through targeted genome methylation—it can be postulated that manipulation of the gametes and subsequent embryo at the very earliest stages of development could impede efficient programming and subsequent organ development.

## 5. Conclusions

The heart is an eminently complex organ, made up of multiple chambers, organized in a three-dimensional conformation allowing multiple axes of asymmetry, made up of different cellular populations with specific functions. To coordinate this, multiple levels of epigenetic regulation allow for precise dosage, temporal and spatial regulation of gene expression. This makes it both vulnerable to perturbations of this epigenetic regulation and intrinsically intolerant to high-impact modifications, as the viability of the embryo is entirely dependent on establishing a functional, beating pump for nutrient and oxygen diffusion. Therefore, the observed spectrum of cardiac heart disease results from a delicate equilibrium between developmental anomaly and viable physiology, in a finite set of possible configurations.

One evident difficulty in fully apprehending the pathophysiology of CHD, once epigenetic regulation becomes a driving motor, is the increasing complexity of disease models. This translates to obvious obstacles in identifying new therapeutic candidates and key regulatory elements; it is now necessary to contend with genic expression, proteic interaction and epigenetic modification of the genome, all extremely dynamic between different timepoints. New research approaches offer an interesting perspective in this specific field: network analysis, integrating the various omics data (genome, epigenome, transcriptome and proteome) [109]. The advantage of such thinking applied to epigenetics in CHD is threefold: 1—reconciling the multiple interplaying elements (DNA modification, RNA interaction, protein recruitment, histone modification, etc.) in an integrated model of interacting regulation levels; 2—the opportunity to create such networks at various temporal and spatial conditions within the developing heart, and therefore being able to unlock key nodes of regulation in critical temporal windows for CHD pathophysiology; and 3—applying machine learning to integrate coregulated nodes intro established models [110], and hereby identifying novel candidates [111].

Progressive unlocking of epigenetic mechanisms also offers a potential field of action to prevent or rescue CHD, the best example of this being the spectacular effect of folic acid supplementation in periconceptional period [85]. To take a bleaker approach, we are in a race against the clock to find efficient prevention techniques, as CHD occurrences seem to trend upwards with the rising prevalence of maternal diabetes, obesity, hypertension and the increasing success of assisted reproduction [112]. So far, DNA demethylating agents or histone deacetylase treatments have been tried out in vitro, but widespread alteration of methylation levels seems a poor solution for what is evidently a targeted dysregulation of gene expression. However, as always when dealing with epigenetics, further discoveries could have a widespread and unexpected impact on our knowledge of physiopathology.

## Figures and Tables

**Table 1:** Overview of documented cardiac manifestations in epigenetic diseases. Cited genes have been proven to have direct regulatory effects on DNA-packing proteins, DNA methylation patterns, DNA conformation.

Epigenetic Function	Syndrome *Affected Gene*	Cardiac Manifestation	Reference
ATP-dependent chromatin modifiers	Coffin–Siris syndrome *ARID1A/B, SMARCB1, SMARCA4, SAMRCE1*	ASD VSD TOF PDA	[40,41,42]
	CHARGE syndrome *CHD7*	TOF DORV VSD AVSD PDA PS IAA	[40,43]
	Sifrim-Hitz-Weiss syndrome *CHD4*	ASD VSD PS PDA TOF MV anomalies	[44]
	Williams syndrome *WSTF*	AS PS	[40]
Histone modifiers	Kabuki syndrome *KMT2D, KDM6A, WDR5*	Coarctation ASD VSD	[40,45,46]
	Kleefstra syndrome *EHMT1*	ASD VSD TOF Coarctation BAV PS	[40,45,47]
	Wolf–Hirschhorn syndrome *WHSC1/2, LETM1*	ASD	[40]
	Rubinstein–Taybi *CREBBP, EP300*	PDA ASD VSD HLHS BAV	[41,45]
	*KAT2B*	ASD VSD PDA TOF PS	[26]
	*PRDM6*	PDA	[27]
	Oculofaciocardiodental syndrome *BCOR*	ASD VSD MV anomalies	[48,49]
Cohesinopathies	Cornelia de Lange *NIPBL, HDAC8, SMC1, SMC3, RAD21, BRD4, ANKRD11*	TOF ASD VSD PDA PS	[22,41]
	Robert’s syndrome *ESCO2*	VSD ASD PDA	[22]
	Warsaw breakage syndrome *DDX11*	TOF VSD	[22]
	ARTX syndrome *ATRX*	VSD ASD TOF PDA PS/AS	[22]
	CHOPS syndrome *AFF4*	VSD PDA	[22]
	STAG2-related X-linked Intellectual Deficiency *STAG2*	VSD	[22]
	CAID syndrome *SGO1*	PS/AS VSD	[22]
Mediatorpathies	Opitz–Kaveggia syndrome *MED12*	TOF	[43,50]
	Lujan–Fryns syndrome *MED12*	TOF	[43,51]
	Ohdo syndrome *MED13L*	TOF	[43]
DNA methylation modulators	ICF syndrome *DMNT3B*	ASD VSD	[40]
Chromatin-modifier regulators	DiGeorge syndrome *TBX1*	IAA Truncus arteriosus TOF TGA VSD	[21,43]

Not included are syndromes involving large DNA rearrangements (i.e., Down syndrome) which are thought to include hits on epigenetic regulators. TOF—tetralogy of Fallot; ASD—atrial septal defect; VSD—ventricular septal defect; TGA—transposition of the great arteries; IAA—interrupted aortic arch; PS—pulmonary stenosis; AS—aortic stenosis; PDA—patent ductus arteriosus; MV—mitral valve; DORV—double-outlet right ventricle.
